# Laparoscopic Resection of Synchronous Liver Metastasis Involving the Left Hepatic Vein and the Common Trunk Bifurcation: A Strategy of Parenchyma-Sparing Resection with Left Sectionectomy and 4a Subsegmentectomy by Arantius Approach

**DOI:** 10.3390/healthcare10030517

**Published:** 2022-03-11

**Authors:** Filippo Banchini, Enrico Luzietti, Gerardo Palmieri, Deborah Bonfili, Andrea Romboli, Luigi Conti, Patrizio Capelli

**Affiliations:** 1 Department of General Surgery, Guglielmo da Saliceto Hospital, 29100 Piacenza, Italy; e.luzietti@ausl.pc.it (E.L.); g.palmieri@ausl.pc.it (G.P.); a.romboli@asul.pc.it (A.R.); l.conti@ausl.pc.it (L.C.); p.capelli@ausl.pc.it (P.C.); 2 Department of General Surgery, Università degli Studi di Parma, 43100 Parma, Italy; deborah.bonfili@gmail.com

**Keywords:** Arantius, hepatic veins, liver metastases, laparoscopy, colorectal liver metastases, minimally invasive liver surgery, colon cancer

## Abstract

When colorectal cancer presents with liver metastasis, hepatic resection remains the most important factor in prolonging survival, and new paradigms have been proposed to augment resectability. An adequate liver remnant and vascularisation are the only limits in complex liver resection, and parenchyma-sparing surgery is a strategy for minimising the complications, preserving liver function, and allowing patients to undergo further liver resection. The laparoscopic approach represents a new challenge, especially when lesions are located in the superior or posterior part of the liver. We discuss the case of an 81-year-old patient with a single synchronous liver metastasis involving the left hepatic vein and leaning into the middle hepatic vein at the common trunk, where we performed a simultaneous laparoscopic colonic resection with a left sectionectomy extended to segment 4a. The strategic approach to the Arantius ligament by joining the left and middle hepatic vein allowed us to avoid a major liver hepatectomy, preserve the liver parenchyma, reduce complications, enhance patient recovery, and perform the entire procedure by laparoscopy. Our example suggests that the Arantius approach to the left hepatic vein and the common trunk could be a feasible approach to consider in laparoscopic surgery for lesions located in their proximity.

## 1. Introduction

Colorectal cancer represents one of the most prevalent oncologic diseases and the third most deadly and fourth most diagnosed cancer worldwide [1]. About 25% of patients present with synchronous metastasis at diagnosis, and about 50% develop metachronous metastasis [2]. It has been reported in the literature that 25% of patients underwent liver resection for metastasis [3], while a population-based analysis showed a rate of 32% [4]. High numbers and sizes of liver metastases are no longer a possible contraindication for resection, so an adequate liver remnant and vascularisation remain the only limiting factors [5]. Multiple strategies for performing major and complex liver resection—from two-stage hepatectomy with portal embolisation [6], the Alpps procedure [7], and enhanced ultrasound-guided liver resection [8], to the conceptual modification of vascular R1 resection with 1 mm free margins—have been developed [9]. Considering these and the multimodality of the treatment of these patients, the tumour biology and the biomarkers KRAS, NRAS, BRAF, TP53, PIK3CA, APC, and mismatch repair deficiency (MMRD) are important factors for prolonging or achieving patient survival [10,11]. A new concept for preserving the liver parenchyma, parenchyma-sparing surgery, has been developed to minimise complications, preserve liver function, and allow patients to undergo further liver resection, which some authors describe as a type of chronic disease [12]. Laparoscopic liver surgery has arisen in this complex scenario, in which a precision-surgery era is developing [13]; however, it remains a challenge, especially for lesions located in difficult positions, and, for this reason, is confined to experienced centres. We present a strategical approach for a single synchronous liver metastasis originating from the left hepatic vein and leaning along the middle hepatic vein—the Arantius approach, within the concept of parenchyma-sparing surgery.

## 2. Case Presentation

**Figure 1:**
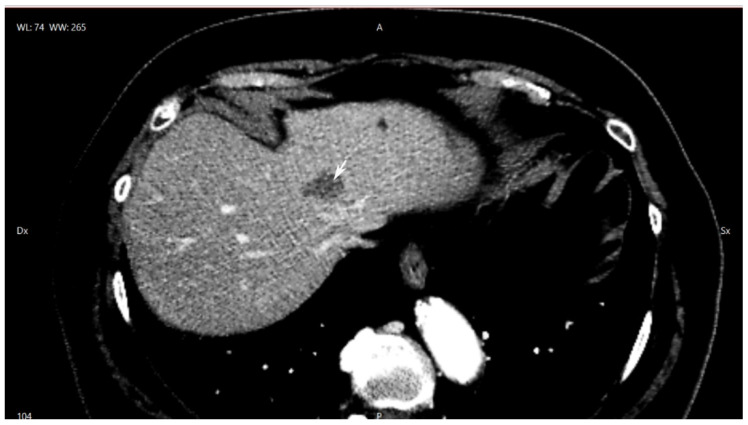
Upper portion of liver CT scan: lesion at the origin of the common hepatic trunk. (white arrow: lesion).

The case is an 81-year-old patient with non-insulin-dependent diabetes, hypertension, and previous intervention for cisto-rectal prolapse. After a positive faecal blood occult test, the patient underwent colonoscopy with evidence of an ulcerated lesion of the right hepatic flexure. A CT scan raised doubt regarding a lesion involving the left hepatic vein in proximity to the bifurcation of the common hepatic trunk (Figure 1). An MRI with liver contrast confirmed the suspicion of liver metastases. After a multidisciplinary discussion, and in agreement with the patient to avoid a double intervention, indication for laparoscopic simultaneous resection was given.

**Figure 2:**
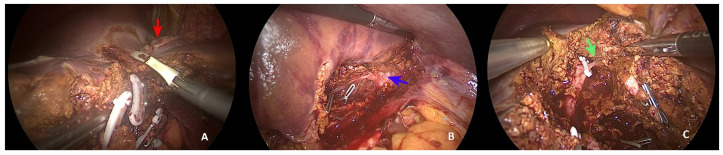
Parenchymal dissection. (**A**) Dissection between segments 4a and 4b (red arrow: liver metastasis); (**B**) exposure of inferior part of common trunk (blue arrow) using the Arantius approach; (**C**) cone unit of segment 4a (green arrow).

The intervention started with the patient in a supine position and abducted legs, to allow simultaneous liver resection and colonic resection. Intraoperative ultrasound showed evidence of a lesion involving the anterior part of the left hepatic vein, including the upper portion of segment 4a, and lying along the last part of the middle hepatic vein up to the common trunk bifurcation. In consideration of the patient’s characteristics and indication for simultaneous resection, a left sectionectomy with en bloc anatomical subsegmentectomy 4a was chosen. The intervention started with the surgeon positioned between the legs. The pedicles of segments 2 and 3 were isolated and ligated, and partial dissection between segments 3 and 4b was performed (Figure 2A).

**Figure 3:**
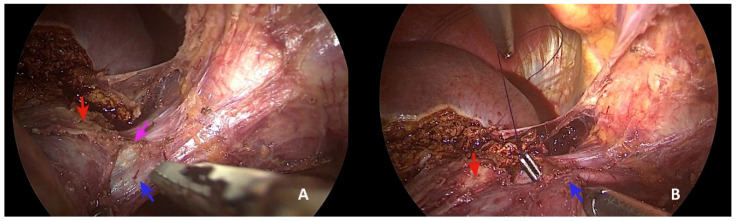
Superior dissection of common hepatic trunk. (**A**,**B**) Exposure of the right margin of the left hepatic vein (blue arrow: left hepatic vein; violet arrow: middle hepatic vein; red arrow: liver metastasis).

Using ultrasound, we marked the liver surface along the middle hepatic vein up to the margin of the liver metastases between segments 4a and 8. The dissection was continued along the Arantius ligament, and the inferior margin of the common hepatic trunk was exposed (Figure 2B). The dissection was continued to join this structure, dissecting the portal branch for segment 4a and identifying a cone unit for the upper part of segment 4a (Figure 2C). Subsequent mobilisation of the left lobe was achieved. The careful dissection of the common trunk from top to bottom was carried out in a craniocaudal manner (Figure 3A).

**Figure 4:**
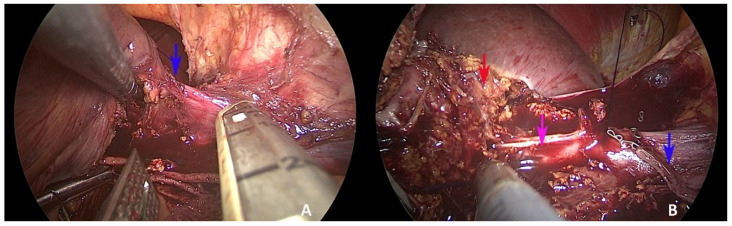
Exposure of the middle hepatic vein. (**A**) Insertion of the linear stapler from the bottom section of the left hepatic vein (blue arrow: left hepatic vein); (**B**) exposure of the middle hepatic vein after section of the left hepatic vein (blue arrow: left hepatic vein; violet arrow: middle hepatic vein; red arrow: liver metastasis).

The subsequent section of the right margin of the left hepatic vein was performed (Figure 3B) using a vascular stapler (Figure 4A). This manoeuvre allowed us to continue the craniocaudal dissection along the middle hepatic vein safely, detaching the lesion from the portion of the middle hepatic vein, as in vascular R1 resection (Figure 4B).

The next part of the intervention was conducted with the surgeon positioned to the left of the patient. The colonoscopy of the marked area evidenced a lesion of the middle transverse colon. The middle colic artery was dissected until its origin at the pancreas, and the right colon was mobilised. Intrabdominal resection was performed with side-to-side colonic anastomosis. The pieces were extracted by Pfannenstiel incision.

Further haemostatic control and drain placement were performed. The total time of intervention was 365 min, and the total blood loss was estimated to be 500 mL. Histopathology revealed T3N0 colon cancer with 20 negative lymph nodes and liver parenchyma with a metastasis of 3 cm × 2 cm with R1 vascular contact. The postoperative course was surgically uneventful, with a liquid diet introduced at j1 and a low-fibre diet in the following days. During the hospitalisation, she had direct contact with a SARS-CoV-2 inpatient, and for the hospital protocol, she was isolated in a separate room and remained asymptomatic; a thoracic CT scan was performed, and she was discharged after 10 postoperative days. The patient is asymptomatic and on a course of chemotherapy.

## 3. Discussion

The resection of liver metastases for colorectal cancer remains the gold standard treatment, combined with chemotherapy. This combination could increase the five-year overall survival from 31% [14] to 66% [15], even with R0–R1 resection. Many different approaches to improving respectability have been taken, such as the liver-first approach [16], two-stage hepatectomy [6], the Alpps procedure [7], and enhanced ultrasound-guided liver resection [8]. Aggressive surgery with resection has also been extended to pulmonary metastases, with three- and five-year survival rates of 67.7% and 39.4%, respectively [17], suggesting that surgery and chemotherapy together constitute a synergic approach to the disease. Multidisciplinary team discussion is becoming essential for achieving high rates of resection, which have reached 41% [15], even in the case of recurrent disease [18]. Considering that more than 50% of the patients developed recurrence, some principles need to be considered for this aggressive strategy to succeed. Knowledge of a given patient’s liver anatomy is essential for approaching resection in a strategical manner, considering probable future repeated surgery, advocating, when possible, the use of a parenchyma-sparing strategy [19] to preserve liver function and the liver parenchyma. Three-dimensional reconstruction could be a useful tool for planning resection, considering the anatomical variations of patients [20,21], facilitating the parenchyma-sparing approach by enabling a high level of precision in surgery [13,22] and exploiting cone unit dissection [23]. Laparoscopic surgery has entered this developing surgery, enabling the use of indocyanine green fluorescence [24] to perform anatomical resection by cone dissection, in combination with high-level technology, to perform complex liver resection [25]. Some gaps remain, particularly for lesions located in the so-called “difficult segment” of the superior portion of the liver, and strategical efforts need to be made to maintain the parenchyma-sparing concept in laparoscopic surgery. The case we presented demonstrates a possible strategy for performing laparoscopic synchronous liver resection and transverse colonic resection by applying the Arantius approach to single metastases presenting in the common trunk of the middle and left hepatic veins. The data in the literature confirm the feasibility of performing simultaneous laparoscopic resection, with less morbidity and shorter postoperative hospital stays [26,27]. The simultaneous approach is considered a very good option for non-rectal primary and peripheral lesions or left lateral sectionectomy [28], but the morbidity could be augmented in the case of major hepatectomy [29], so careful patient selection is recommended for synchronous major hepatectomy [27]. Based on the characteristics of our patient, in the interest of reducing the morbidity and preventing postoperative liver failure in the case of colonic anastomotic complications, we decided to minimise the liver resection instead of performing a left hepatectomy. The adopted strategy allowed us to apply parenchyma-sparing surgery to prevent postoperative liver failure and minimise complications. In the era of enhanced recovery after surgery, the laparoscopic approach has been demonstrated to improve the outcomes of patients [30], and this approach needs to be considered even in simultaneous resection. In conclusion, the Arantius approach for a metastasis located in an awkward position allows one to apply a modern laparoscopic approach with the preservation of the liver parenchyma and a precise and anatomical dissection, preventing postoperative morbidity and facilitating an early recovery of the patient.

## 4. Conclusions

Laparoscopic resection is an emerging strategy in liver resections. Combining the concepts of open surgery and laparoscopy is essential for proceeding in performing liver surgery, with the application of parenchyma-sparing strategy and precise dissection having the advantage of reducing postoperative morbidity in an enhanced recovery program. The Arantius approach to the left hepatic vein and the common trunk could be a feasible approach to consider in laparoscopic surgery for lesions located in their proximity.

## Data Availability

The study did not report any data.
